# Microbisporicin (NAI-107) protects *Galleria mellonella* from infection with *Neisseria gonorrhoeae*

**DOI:** 10.1128/spectrum.02825-23

**Published:** 2023-10-12

**Authors:** Nele Hofkens, Zina Gestels, Said Abdellati, Irith De Baetselier, Philippe Gabant, Anandi Martin, Christopher Kenyon, Sheeba Santhini Manoharan-Basil

**Affiliations:** 1 Department of Clinical Sciences, Institute of Tropical Medicine Antwerp, Antwerp, Belgium; 2 Clinical Reference Laboratory, Department of Clinical Sciences, Institute of Tropical Medicine Antwerp, Antwerp, Belgium; 3 Syngulon, Seraing, Belgium; 4 Department of Medicine, University of Cape Town, Cape Town, South Africa; Griffith University - Gold Coast Campus, Southport, Gold Coast, Queensland, Australia

**Keywords:** *Neisseria gonorrhoeae*, lantibiotics, NAI-107, microbisporicin, *Galleria mellonella*

## Abstract

Due to the increasing incidence of antibiotic-resistant bacterial pathogens, the need for the development of alternative compounds is of utmost importance. Bacteriocins are considered a promising alternative to antibiotics. In this study, we determined the *in vitro* activities of 65 bacteriocins from the PARAGEN collection along with microbisporicin (NAI-107) against 14 WHO reference isolates of *Neisseria gonorrhoeae*, one isolate of *Neisseria meningitidis*, and four commensal *Neisseria* spp. The initial screening of the bacteriocins was carried out using a spot-on-lawn assay wherein 12 bacteriocins (12/66) showed a zone of inhibition against the WHO-P *N. gonorrhoeae* isolate. Four bacteriocins (4/12), Garvicin KS (GarKS) (ABC), lacticin (lcn) Q, lcn Z, and NAI-107, demonstrated higher activity, and minimum inhibitory concentrations (MICs) using agar dilutions were determined for 19 *Neisseria* spp. GarKS (ABC), lcn Q, and lcn Z had an MIC range of 4–16 µg/mL, whereas NAI-107 had an MIC range of <0.25 to 2 µg/mL. Using an *in vivo Galleria mellonella* model, we determined the efficacy of GarKS (ABC), lcn Z, and NAI-107. GarKS (ABC) and lcn Z displayed no *in vivo* toxicity but showed no *in vivo* activity against WHO-P *N. gonorrhoeae*. In contrast, NAI-107 was as effective as ceftriaxone and significantly increased the survival rate of WHO-P *N. gonorrhoeae*-infected *G. mellonella* larvae. In conclusion, NAI-107 exhibited potent *in vitro* and *in vivo* activities against *N. gonorrhoeae*. To our knowledge, this is the first study to demonstrate that NAI-107 possesses anti-gonococcal activity *in vivo*.

**IMPORTANCE:**

We screened 66 bacteriocins to see if they exhibited anti-gonococcal activity. We found 12 bacteriocins with anti-gonococcal effects, and 4 bacteriocins showed higher anti-gonococcal activity. Three bacteriocins, lacticin Z, lacticin Q, and Garvicin KS (ABC), showed *in vitro* anti-gonococcal activity but no *in vivo* inhibitory effects against the *Neisseria gonorrhoeae* (WHO-P) isolate. On the other hand, NAI-107 showed *in vivo* anti-gonococcal activity. The findings suggest that NAI-107 is a promising alternative to treat gonorrhea infections.

## INTRODUCTION

The emergence, spread, and persistence of multidrug-resistant (MDR) bacteria pose a global threat of growing concern to human, animal, and environmental health. The scarcity of new antibacterial drugs in the development pipeline exacerbates the serious threat to public health posed by the spread of antibiotic resistance, which is forecast to reach dramatic levels by 2050 (1 – 4). The majority of antibiotics used in clinical practice today are microbial metabolites or their derivatives, and alternative approaches to antibiotic discovery have not been as successful as microbial product screening (5, 6). The increasing incidence of antibiotic-resistant bacterial pathogens highlights the urgent need for new antimicrobial agents, especially those with new modes of action (2).

Gonorrhea caused by the pathogen *Neisseria gonorrhoeae* is the second most common bacterial sexually transmitted infection (STI). In 2020, the World Health Organization (WHO) estimated that 82.4 million new gonorrhea cases occurred in adults globally (7). Complications of gonorrhea include pelvic inflammatory disease, infertility, and ectopic pregnancy in women, epididymal orchitis in men, neonatal ophthalmia, and an elevated risk for HIV acquisition and transmission (8 – 11). The increased emergence of antimicrobial resistance (AMR) in *N. gonorrhoeae* has heightened the need for new first-line antimicrobials for the treatment of gonorrhea (11).

Bacteriocins are antimicrobial peptides produced by bacteria with either a broad or narrow spectrum of antibacterial activity (12 – 14). According to the Cotter et al. scheme, bacteriocins are classified into lanthionine (class I) and non-lanthionine-containing (class II) bacteriocins (15). Lantibiotics (class I) are ribosomally synthesized, post-translationally modified peptides (RiPPs) (16). They are small (<5 kDa) peptides and are synthesized as a precursor peptide, lanthionine (LanA), with an N-terminal leader peptide (NP) and a C-terminal core peptide (CP) (17). They undergo post-translational modifications (PTMs) with the formation of 3-methyllanthionine (MeLan), dehydroalanine (Dha), and/or dehydrobutyrine (Dhb) residues that occur *via* the dehydration of serine and threonine residues, respectively, which are then crosslinked *via* a thioether linkage with cysteine residues and a number of dehydrated amino acids that gives lantibiotics their characteristic conformation and stability (15 – 22). Examples include microbisporicin (NAI-107) produced by the actinomycete *Microbispora corallina* ATCC-PTA-5024 (23 – 28) and nisin Z from *Lactococcus lactis* IO-1 (29). Class II bacteriocins are the most frequent naturally occurring bacteriocins. They are small (<10 kDa), are heat-stable, and, unlike lantibiotics, do not undergo extensive post-translational modification. Examples include lacticin Z (lcn Z) and lacticin Q (lcn Q) produced by *L. lactis* QU 14 and QU 5, respectively (30, 31), and Garvicin KS (GarKS) produced by *Lactococcus garvieae* KS1546 (32).

Bacteriocins have gained considerable attention as potential alternatives to traditional antibiotics due to their narrow-spectrum activity and a lower likelihood of inducing resistance (12 – 14, 33). Most leaderless bacteriocins, such as lacticin Q (53 aa), lacticin Z (53 aa), and Garvicin KS [composed of three peptides (GakA, GakB, and GakC, 30–34 aa)], have been shown to have inhibitory effects against a range of genera, such as *Bacillus*, *Listeria*, *Staphylococcus*, and *Enterococcus* (32, 34). NAI-107 has been found to be active against a wide range of MDR Gram-positive bacterial pathogens, including methicillin-resistant *Staphylococcus aureus* (MRSA), glycopeptide-intermediate-resistant *S. aureus* (GISA), vancomycin-resistant enterococci (VRE), penicillin-resistant *Streptococcus pneumoniae*, and a selected range of Gram-negative bacteria*—N. gonorrhoeae* and *Moraxella* spp. (24, 33, 35). .

*Galleria mellonella* larvae have been previously used as a model host for studying various pathogens and the efficacy of various antimicrobial agents (36 – 43). This system provides an alternative *in vivo* model to mammalian systems, and its immune system involves both cellular (phagocytosis, nodulization, and encapsulation) and humoral defenses (melanization, hemolymph clotting, and production of antimicrobial peptides) and shares a high degree of structural and functional similarity with that of mammals (36).

The *in vitro* antimicrobial activity of bacteriocins against relevant pathogenic bacteria (including MDR pathogens) has been well documented (44 – 47). However, the use of *in vivo* studies is an important step forward for the development of new therapeutic agents (48). The present study investigated the *in vitro* antibacterial activity of lantibiotics (*n* = 66) and the *in vivo* antibacterial activity of three bacteriocins against WHO-P *Neisseria gonorrhoeae* using the wax moth larva *G. mellonella*. The results highlight the potential of bacteriocins as alternative therapeutic agents for treating *N. gonorrhoeae* infections.

## MATERIALS AND METHODS

### Bacterial isolates

The following isolates were used in the present study: *N. gonorrhoeae* WHO reference isolates (*n* = 14), *N. meningitidis* (*n* = 1, ITM 3354), *Neisseria mucosa* (*n* = 1, ATCC 25999), *Neisseria subflava* (ITM 3367), *Neisseria cinerea* (ATCC 14685), *Neisseria lactamica* (*n* = 1, ATCC 23970), and *L. lactis* subsp. lactis IL1403 (*n* = 1) (Table 2). In Belgium, resistance to gonococcal azithromycin has increased from 0.2% to 18.6% over the past 8 years, despite the use of dual therapy, i.e., ceftriaxone and azithromycin (49). Therefore, in this study, the WHO-P *N. gonorrhoeae* isolate was used in the initial *in vitro* screening and *in vivo* experiments due to its susceptibility to ceftriaxone (0.004 µg/mL) and resistance to azithromycin (4 µg/mL), as determined by the European Committee on Antimicrobial Susceptibility Testing (EUCAST) criteria (50).

### *In vitro* studies

#### Source of bacteriocins and determination of the anti-gonococcal activity of bacteriocins

Sixty-five bacteriocins from the PARAGEN synthetic DNA library collection from the Belgian start-up Syngulon (51) and NAI-107 (AdipoGen Life Sciences) were used in the study. The stock of each bacteriocin from PARAGEN was prepared in molecular biology-grade water (AccuGENE). NAI-107 was dissolved in 15% dimethyl sulfoxide (DMSO). The bacteriocins were stored at −20°C until further use. The antibacterial activities of the bacteriocins against the azithromycin-resistant WHO-P *N. gonorrhoeae* isolate were tested by spot-on-lawn assay. Briefly, the direct colony suspension method was used to grow the WHO-P isolate, wherein the inoculum was prepared using phosphate-buffered saline (PBS) from isolated colonies grown overnight on a gonococcal (GC) [containing 15 g/L Bacto protease peptone, 1 g/L soluble starch, 4 g/L K_2_HPO_4_ (174.18 g/mol), 1 g/L KH_2_PO_4_ (136.08 g/mol), and 5 g/L NaCl (58.44 g/mol), supplemented with 1% BD BBL IsoVitaleX] agar plate. The turbidity of the suspension was adjusted to a 0.5 McFarland standard. Specifically, 100 µL of the inoculum suspension was added to 5 mL of GC soft agar, mixed, and overlaid on the surface of the GC agar plate and allowed to solidify. Two microliters of the bacteriocin was spotted on the surface. Plates were incubated in a 5% CO_2_ incubator at 37 ^o^ C for 24 hours. The antimicrobial activity was evaluated based on the zone of inhibition (ZOI). Based on the size of the ZOI, a select number of antimicrobial bacteriocins were used to determine the minimum inhibitory concentration (MIC).

#### Minimum inhibitory concentrations of lacticin Z, lacticin Q, GarKS ABC, and NAI-107 against *Neisseria* species

The MICs were determined using the agar dilution method based on the Clinical and Laboratory Standards Institute (CLSI) for the four bacteriocins (lacticin Z, lacticin Q, GarKS ABC, and NAI-107) against 19 *Neisseria* spp. (52). *L. lactis* IL1403, for which the PARAGEN collection bacteriocins showed antibacterial activity by spot assays, was used as a positive control (53).

In brief, for the agar dilution, a series of GC agar plates with 1% IsoVitaleX containing the bacteriocin to be tested in increasing concentrations in doubling dilutions (i.e., 2, 4, 8, and 16 µg mL^−1^ for lacticin Z, lacticin Q, and GarKS ABC and 0.25, 0.5, 1, 2, and 4 µg mL^−1^ for NAI-107) were prepared. The strains were suspended in Mueller-Hinton (MH) broth to equal the turbidity of a 0.5 McFarland standard [∼1 × 10^8^ colony forming units (CFU) mL^−1^], and 200 µL of one-tenth dilution of the suspension was placed on the inoculum plate. The inoculum was transferred using an AQS A400 Multipoint Inoculator (the final inoculum is ∼10^3^ CFU/spot). Blood agar and GC agar plates with no bacteriocin added served as the growth control plates. The agar plates were incubated at 37°C for 18–24 hours in 5% CO_2_. After incubation, the MIC was considered the lowest concentration of the tested material that inhibited the visible growth of the bacteria.

#### Serial passage experiments and evaluation of the potential for *in vitro* resistance development to NAI-107

*N. gonorrhoeae* WHO-P was propagated by serial passages with increasing concentrations of NAI-107 (0.25, 0.5, 1, 2, and 4 µg/mL) in quadruplicate. The selection of WHO-P was performed on GC agar plates supplemented with various concentration gradients of NAI-107 with an initial low sub-MIC concentration (one-fourth of MIC) of 0.25 µg/mL and incubated at 37°C in 5% CO_2_. After visible growth was attained, colonies were subcultured onto GC agar plates with twofold serial bacteriocin concentrations. The serial passage was repeated until no visible growth was seen on the GC agar plate.

### *In vivo* studies

#### Co-culturing of *N. gonorrhoeae* with the hemolymph from *G. mellonella*

The *N. gonorrhoeae* inoculum for infection in *G. mellonella* was prepared as follows: the WHO-P *N. gonorrhoeae* isolate was cultured from frozen stocks onto BD Columbian Blood Agar with 5% sheep blood for 24 hours at 37°C with 5% (v/v) CO_2_. Single colonies were plated onto fresh BD chocolate blood agar plates and incubated. A loopful of the culture from the agar plates was suspended in PBS containing the hemolymph from *G. mellonella* larvae. The mixture was incubated at 37°C with 5% (v/v) CO_2_ for an hour and plated on fresh BD BBL GC-lect selective agar to enrich the growth and recovery of *N. gonorrhoeae* specifically. This inoculum was used to infect *G. mellonella* subsequently.

#### Determining the maximum nonlethal dose of *N. gonorrhoeae*

*G. mellonella* larvae were treated with a range of inoculating doses of *N. gonorrhoeae* WHO-P (1.5 × 10^8^–1.2 × 10^9^ CFU/mL) and observed for death alongside PBS controls. The maximum nonlethal dose was determined and used as the inoculating dose for infection. The nonlethal dose with 80% mortality was observed at concentrations as high as 9.0 × 10^8^ CFU/mL and was used in the *G. mellonella* model.

#### *Galleria mellonella* infection model

Injection of *G. mellonella* was carried out as described in reference (43). The last larval stage of *G. mellonella* (Terramania, Arnhem, Netherlands) was used for the experiments. Only macroscopically healthy, non-discolored larvae were selected. The larvae were inoculated in the last left proleg using 0.3-mL U-100 insulin syringes (BD Micro-Fine). Two test groups were evaluated: (i) to assess the efficacy, test group 1 consisted of 20 larvae per concentration of each bacteriocin or ceftriaxone (20 mg/kg). About 30 µL of the 3.0-McFarland (2.7 × 10^7^ CFU/mL) *N. gonorrhoeae* inoculum in PBS was used to infect *G. mellonella* larvae. The larvae were treated with 1× MIC, 2× MIC, 4× MIC, and 8× MIC of GarKS (ABC) and lacticin Z and 4× MIC, 8× MIC, and 16× MIC of NAI-107; (ii) to assess the toxicity, test group 2 with five larvae per concentration of bacteriocin was used. The efficacy test group 1 first received the *N. gonorrhoeae* inoculum, followed by the bacteriocin or antimicrobial, whereas the toxicity test group 2 received only the bacteriocin. One positive and two negative control groups were used. The positive control group received only the *N. gonorrhoeae* inoculum in PBS. Out of the two negative control groups, one group underwent no manipulation, while the other group was injected with PBS only. Each group was incubated in sterile Petri dishes at 37°C with a 5% (v/v) CO_2_ atmosphere for the duration of the experiments. The larvae were observed for 120 hours for any indications of illness, necrosis, or paralysis, which enabled an evaluation of the bacteriocin’s toxicity. Larvae were scored dead if they did not respond to touch stimuli by blunt sterile forceps and scored for five consecutive days (120 hours).

**Fig 1:**
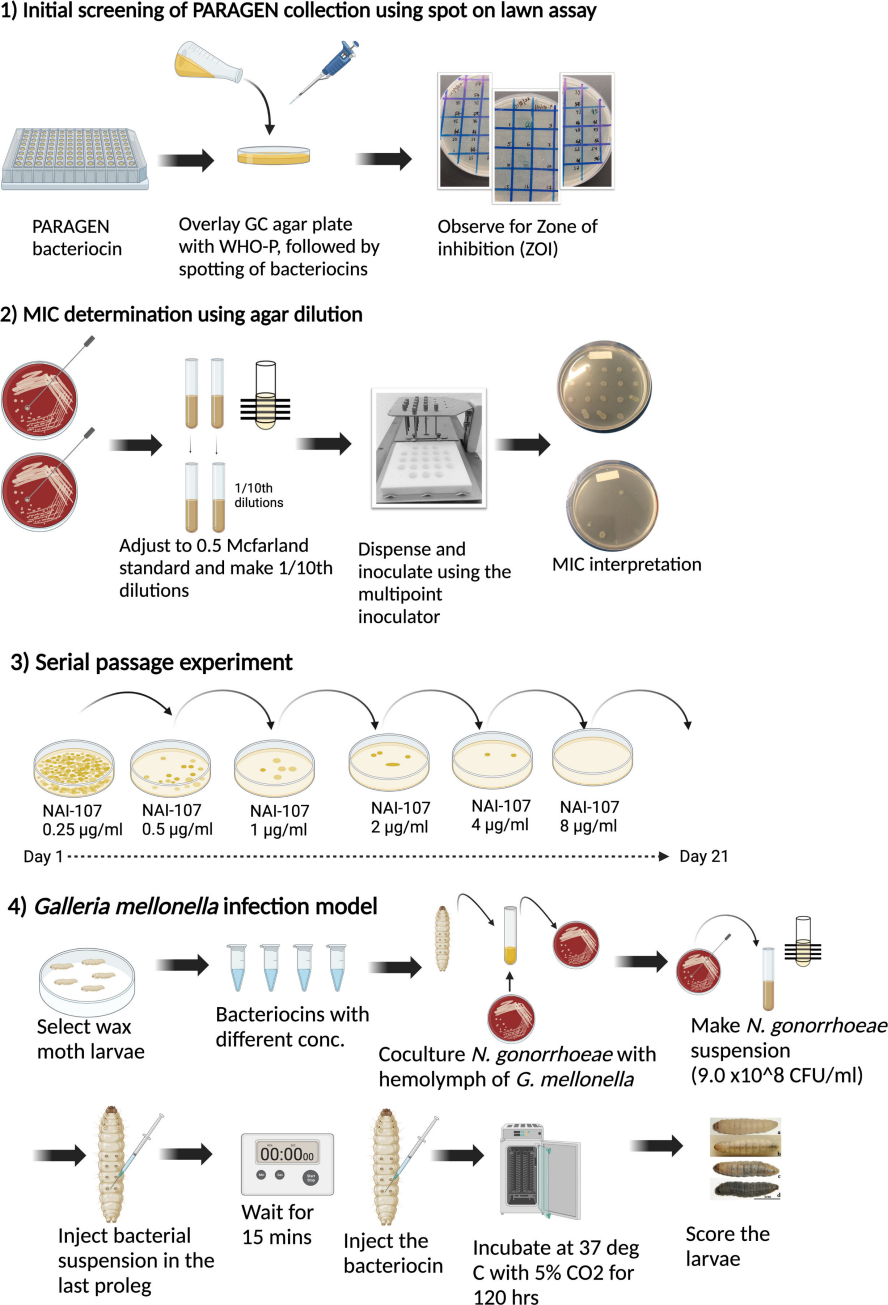
Overview of the study. The figure was created using BioRender.

An overview of the study is provided in Fig. 1.

### Statistical analysis

Data were analyzed using GraphPad Prism v9. Survival plots were created using Kaplan-Meier survival curves. Statistical analysis was carried out using the Mantel-Cox test to compare survival curves between the PBS controls and each treatment arm. A *P* value of <0.05 was considered statistically significant.

Pearson’s correlation coefficient was determined for NAI-107 vs ceftriaxone and NAI-107 vs azithromycin. A Pearson’s correlation coefficient (*r*) of 1 indicated a perfect direct relationship, an *r* value of −1 indicated a perfect inverse relationship, and an *r* value of 0 indicated no relationship between the two variables. Statistical analysis was performed using RStudio (2022.07.1).

## RESULTS

### *In vitro* anti-gonococcal activity of bacteriocins and MICs

Using the spot-on-lawn assay, 66 bacteriocins were screened, and 13 bacteriocins (PARAGEN collection, *n* = 12), including NAI-107, showed anti-gonococcal activity against the WHO-P strain. The sequences and the activity of the bacteriocins are provided in Table 1.

**TABLE 1:** List of bacteriocins with anti-gonococcal activity detected in this study, their producer organisms, amino acid sequences, and *in vitro* activity against *N. gonorrhoeae* WHO-P^*a*^

Bacteriocin	Abbreviation	Producer	Length (aa)	Amino acid sequence	Spot assay (zone of inhibition)
Garvicin KS - A	GarKS-A+B+C	*Lactococcus garvieae*	34	MGAIIKAGAKIVGKGVLGGGASWLGWNVGEKIWK	+++
Garvicin KS - C			32	MGAIIKAGAKIVGKGALTGGGVWLAEKLFGGK	
Garvicin KS - B			34	MGAIIKAGAKIIGKGLLGGAAGGATYGGLKKIFG	
Lacticin Z	LcnZ	*L. lactis*	53	MAGFLKVVQILAKYGSKAVQWAWANKGKILDWINAGQAIDWVVEKIKQILGIK	+++
Lacticin Q	LcnQ	*L. lactis*	53	MAGFLKVVQLLAKYGSKAVQWAWANKGKILDWLNAGQAIDWVVSKIKQILGIK	+++
Acidocin LF221B	Acd221B	*Lactobacillus gasseri*	48	NKWGNAVIGAATGATRGVSWCRGFGPWGMTACALGGAAIGGYLGYKSN	++
Weissellicin M	WeisM	*Weissella hellenica*	43	MVSAAKVALKVGWGLVKKYYTKVMQFIGEGWSVDQIADWLKRH	++
Lacticin Z_C*	LcnZ_C*	*L. lactis*	48	MLAFLKLVAKLGPKAAKWAWANKSRVLGWIRDGMAIEWIINKINDIVN	+
Aureocin A53	AurA53	*S. aureus*	51	MSWLNFLKYIAKYGKKAVSAAWKYKGKVLEWLNVGPTLEWVWQKLKKIAGL	+
Enterocin 7A	Ent7A+B	*Enterococcus faecium*	44	MGAIAKLVAKFGWPIVKKYYKQIMQFIGEGWAINKIIDWIKKHI	+
Enterocin 7B			43	MGAIAKLVAKFGWPFIKKFYKQIMQFIGQGWTIDQIEKWLKRH	
Enterocin L50A	EntL50A+B	*E. faecium*	44	MGAIAKLVAKFGWPIVKKYYKQIMQFIGEGWAINKIIEWIKKHI	+
Enterocin L50B			43	MGAIAKLVTKFGWPLIKKFYKQIMQFIGQGWTIDQIEKWLKRH	
Mutacin BHTB	MutBHTB	*Streptococcus rattus*	44	MWGRILAFVAKYGTKAVQWAWKNKWFLLSLGEAVFDYIRSIWGG	+
Microbisporicin (prepropeptide)	NAI-107	*M. corallina*	57	MPADILETRTSETEDLLDLDLSIGVEEITAGPAVTSWSLCTPGCTSPGGGSNCSFCC	+++

^*a*^ Low (+), moderate (++), and high (+++) anti-gonococcal activities of bacteriocins are shown. Bacteriocins with +++ activity were further chosen for MIC determination.

Four bacteriocins, GarKS (ABC), lacticin Q, lacticin Z, and NAI-107, showed higher activity against WHO-P, and their MICs were determined for 19 *Neisseria* isolates. The MIC of the four bacteriocins against these 19 isolates is provided in Table 2. NAI-107 showed an extended antimicrobial spectrum and was highly active against all *N. gonorrhoeae* isolates. Against all 14 tested *N. gonorrhoeae* isolates, the MICs ranged from 0.25 to 2 µg/mL, with no correlation with the isolate’s ceftriaxone or azithromycin susceptibility profiles. The MIC of *N. meningiditis* was 4 µg/mL, and the MIC range of other *Neisseria* spp. was from 1 to >4 µg/mL (Table 2). In contrast, the other bacteriocins, GarKS (ABC) (MIC range 4 to >16 µg/mL), lacticin Q (MIC range 16 to >16 µg/mL), and lacticin Z (MIC range 8 to >16 µg/mL), were substantially less active than NAI-107 against *Neisseria* species. GarKS (ABC), lacticin Q, and lacticin Z showed some activity against *N. gonorrhoeae* isolates but were inactive against *N. gonorrhoeae* WHO-N, *N. meningiditis,* and *Neisseria* commensals at the highest concentration tested (16 µg/mL) (Table 2).

**TABLE 2:** Minimum inhibitory concentrations (MICs) of GarKS (ABC), lacticin Q, lacticin Z, and NAI-107 determined using the agar dilution method against 19 *Neisseria* spp., including *L. lactis* as a positive control^*a*^

*S. no.*	Bacterial species	Strains	GarKS (ABC) (µg/mL)	Lacticin Q (µg/mL)	Lacticin Z (µg/mL)	NAI-107 (µg/mL)	Ceftriaxone (µg/mL)	Azithromycin (µg/mL)
1	*N. gonorrhoeae*	WHO G	8	16	8	1	0.008	0.25
2		WHO K	8	16	8	<0.25	0.064	0.25
3		WHO M	8	16	8	0.5	0.016	0.25
4		WHO L	16	16	16	1	0.25	0.5
5		WHO N	>16	>16	>16	2	0.004	0.25
6		WHO O	8	16	8	2	0.032	0.25
7		WHO P	8	16	8	1	0.004	4
8		WHO U	4	16	8	0.5	0.002	4
9		WHO V	16	16	8	2	0.064	>256
10		WHO W	8	16	8	1	0.064	0.5
11		WHO F	8	16	8	2	<0.002	0.125
12		WHO X	8	16	8	1	2	0.5
13		WHO Y	>16	>16	8	0.5	1	1
14		WHO Z	4	16	8	1	0.5	1
15	*N. meningitidis*	ITM-3354	>16	>16	>16	4	nd	nd
16	*Neisseria commensals*	*N. lactamica*	>16	16	16	1	nd	nd
17		*N. mucosa*	>16	>16	16	>4	nd	nd
18		*N. subflava*	>16	>16	>16	4	nd	nd
19		*N. cinerea*	>16	>16	16	2	nd	nd
20		*L. lactis*	<2	16	4	<0.25	nd	nd

^*a*^ Ceftriaxone and azithromycin MICs have been adapted from reference (50). nd, not determined. For NAI-107 vs ceftriaxone, Pearson’s correlation (*r*) = −0.207, *P* value = 0.477, and for NAI-107 vs azithromycin, *r* = 0.396, *P* value = 0.159.

### *In vivo* toxicity of bacteriocins in *G. mellonella*

**Fig 2:**
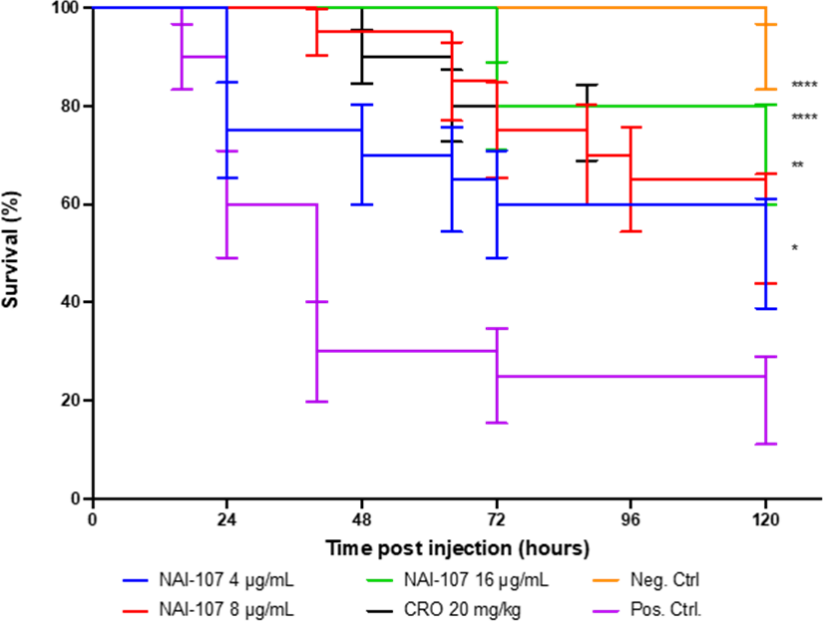
*In vivo* efficacy (survival) of NAI-107 and ceftriaxone (20 mg/kg) against *N. gonorrhoeae,* the WHO-P reference strain in *G. mellonella* larvae. The test groups were injected with 2.7 × 10^7^ CFU/mL of *N. gonorrhoeae*, the WHO-P strain in PBS, followed by NAI-107 (doses 4, 8, and 16 µg/mL) or ceftriaxone (20 mg/kg). The negative control group was injected with 30 µL of PBS, and the positive control group was injected with 2.7 × 10^7^ CFU/mL of *N. gonorrhoeae* in PBS. Both the test and control groups consisted of 20 larvae and were incubated for 120 hours at 37°C. Error bars represent the standard errors. Asterisks represent the significance levels based on the *P* values comparing the survival curves between the PBS-positive controls and each treatment arm (Mantel-Cox test). A single asterisk (*) denotes a *P* value between 0.01 and 0.09. Two (**), three (***), and four (****) asterisks represent a *P* value in the range of 0.001–0.009, 0.0001–0.0009, and <0.0001, respectively.

GarKS (ABC), lacticin Z, and NAI-107, which were active against the *N. gonorrhoeae* WHO-P isolate, were chosen for the *in vivo* experiments. GarKS (ABC), lacticin Z, and NAI-107 administered at doses up to 64 and 16 µg/mL, respectively, did not result in the death of the larvae (Fig. 2; Fig. S1a and b).

### NAI-107 can protect *G. mellonella* larvae from infection with WHO-P *N. gonorrhoeae*

The *in vivo* efficacy of GarKS (ABC), lacticin Z, and NAI-107 was determined against *N. gonorrhoeae* WHO-P in comparison to ceftriaxone. Despite displaying *in vitro* activity against *N. gonorrhoeae,* the two bacteriocins, GarKS (ABC) and lacticin Z, did not demonstrate any detectable efficacy *in vivo* (Fig. S1a and b). NAI-107 was effective *in vivo* at all doses tested (*P* values 0.01 to <0.0001). A small dose-response effect may have been present, with mortality being lowest at the highest dose tested (16 µg/mL) and increasing stepwise at decreasing doses. At the two highest doses tested (8 and 16 µg/mL), the survival of *G. mellonella* was similar to that of the ceftriaxone group (Fig. 2).

### Lack of *in vitro* resistance in *N. gonorrhoeae*

After subjecting *N. gonorrhoeae* WHO-P to four consecutive subcultures for 21 days in the presence of doubling concentrations of NAI-107, it resulted in a modest increase in the NAI-107 MIC, specifically a twofold increase (4 µg/mL).

## DISCUSSION

Previously, microbisporicin (NAI-107) was reported to have *in vitro* efficacy against *N. gonorrhoeae* (33). Using a spot-on-lawn assay, we screened 65 bacteriocins from the PARAGEN collection along with NAI-107 against WHO-P *N. gonorrhoeae* (33, 51). Out of the 65 PARAGEN bacteriocins, a zone of inhibition was observed for 12 bacteriocins, and 3 bacteriocins showed the highest *in vitro* activity (Table 2). The *N. gonorrhoeae* MIC values of three PARAGEN bacteriocins, GarKS (ABC) (MIC range 4 to >16 µg/mL), lacticin Q (MIC range 16 to >16 µg/mL), and lacticin Z (MIC range 8 to >16 µg/mL), were higher than those for NAI-107 (MIC range <0.25 to 2 µg/mL). This MIC range was similar to that reported by the study of Brunati et al. (33), where the MIC range of *N. gonorrhoeae* (*n* = 18) isolates with intermediate or high resistance to penicillin had MICs that ranged from 0.015 to 2 µg/mL. Interestingly, there was no apparent correlation between the MIC of NAI-107 and *N. gonorrhoeae*’s ceftriaxone and azithromycin susceptibility profiles. In particular, the isolates with high ceftriaxone MICs had low NAI-107 MICs. This, in conjunction with its similar half-life to ceftriaxone, makes it an attractive option for combination therapy to prevent the further emergence of ceftriaxone resistance (33). Our *in vivo* tests in *G. mellonella* confirmed the therapeutic efficacy and lack of toxicity of NAI-107. Finally, the *in vitro* passage experiments showed only a modest increase in the NAI-107 MIC; specifically, a twofold increase in MICs was observed. The same was observed in Gram-positive organisms, such as *S. aureus,* and interestingly, no spontaneous resistant mutants were observed (33, 54)

In contrast to NAI-107, the two PARAGEN bacteriocins [GarKS (ABC) and lacticin Z] did not demonstrate *in vivo* efficacy against *N. gonorrhoeae*; however, they showed no *in vivo* toxicity. The observed disparity between the *in vitro* and *in vivo* activities of the PARAGEN bacteriocins suggests the presence of additional factors influencing their efficacy *in vivo*. Several possibilities warrant consideration. Little is known about the pharmacodynamics and pharmacokinetics of these bacteriocins in the *G. mellonella* infection model (55 – 57). It is possible, for example, that NAI-107 is the only bacteriocin assessed that is not metabolized by the host. Various modes of action have been postulated for leaderless bacteriocins. Generally, leaderless bacteriocins have been shown not to involve a receptor/docking molecule to exhibit their antimicrobial action (46).

For instance, lacticin Q forms a huge toroidal pore (HTP) in the bacterial cell membrane, is translocated beneath the cell membrane, does not require a docking molecule, and is thus highly dependent on the physiological features of the cell membrane. This is thought to partly explain its limited spectrum of antibacterial activity and selective antimicrobial action (58, 59). The leaderless aureocin A53 produced by *S. aureus* A53 shows a stronger interaction with neutral membranes than negatively charged lipids and permeates the bacterial membranes without forming pores (60). In another instance, the leaderless bacteriocin LsbB, isolated from *L. lactis* subsp. *lactis* BGMN1–5, requires a zinc-dependent membrane metallopeptidase, YvjB, as its receptor molecule, which is contrary to the general understanding that leaderless bacteriocins do not require a receptor molecule for their mode of action (46, 61). The lantibiotic NAI-107, unlike nisin, does not form stable pores but causes depolarization of the membrane, resulting in bacterial cell death (28). It is thus plausible that the absence of a signal peptide and, in general, various modes of action of the leaderless peptides affects their processing, localization, or recognition by immune cells, leading to reduced efficacy *in vivo* (46). Further research is warranted to elucidate the underlying mechanisms responsible for the reduced *in vivo* activity of leaderless peptides, such as PARAGEN bacteriocins. Investigating these peptides’ stability, secretion pathways, and host interactions could provide valuable insights into their efficacy and pave the way for their optimization as therapeutic agents.

The contrasting results observed for NAI-107 and the PARAGEN bacteriocins raise other interesting questions. While *in vitro* studies provide valuable initial insights, they may not fully reflect the complexity of the *in vivo* environment.

The study’s caveats include a lack of testing in a mammalian model of *N. gonorrhoeae*. Notably, NAI-107 has been shown to be effective against methicillin-resistant *S. aureus* in a rat model (62). While no evidence of toxicity was detected in this study, further studies are required to assess toxicity in other animal models before NAI-107 can be tested in humans. Further testing should assess if there is evidence of synergy or antagonism between NAI-107 and ceftriaxone and other potential combination therapies.

Despite the above shortcomings, the results of the current work indicate that NAI-107 represents a promising option for addressing the severe threat of antibiotic resistance in *N. gonorrhoeae* and possibly in *N. meningitidis*.

## SUPPLEMENTAL MATERIAL

Supplemental Figure S1 spectrum.02825-23-s0001.docx Supplemental Figure S1a and b, showing two Kaplan-Meier plots.

ASM does not own the copyrights to Supplemental Material that may be linked to, or accessed through, an article. The authors have granted ASM a non-exclusive, world-wide license to publish the Supplemental Material files. Please contact the corresponding author directly for reuse.
