# Supplementary material for: Microbisporicin (NAI-107) protects Galleria mellonella from infection with Neisseria gonorrhoeae
Source: Microbiol Spectr. 2023 Oct 12;11(6):e02825-23. doi: 10.1128/spectrum.02825-23 (PMC10715042; doi:10.1128/spectrum.02825-23)
Supplement: Supplemental Figure S1 — a and b, showing two Kaplan-Meier plots. [file spectrum.02825-23-s0001.docx]

**(a)**


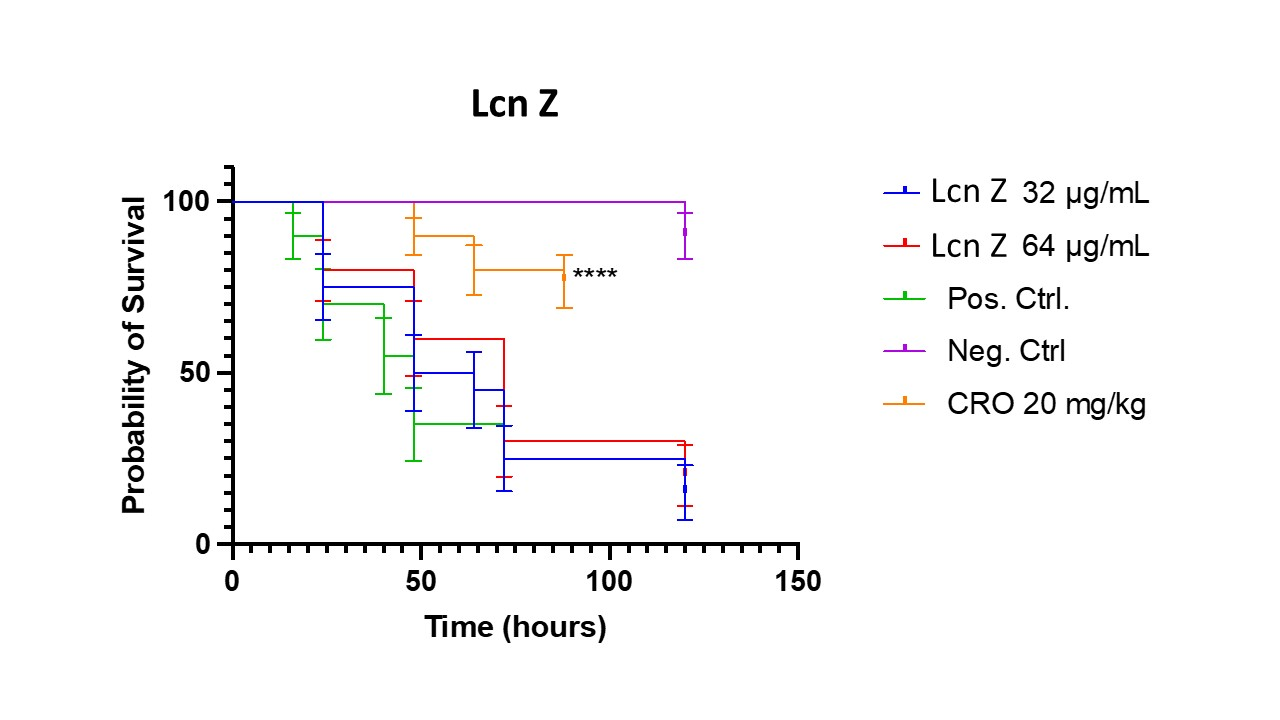


**(b)**

**Supplementary Figure 1:** (a) *In vivo* efficacy (survival) of lacticin Z (Lcn Z), (b) Garvicin KS (GarKS) and ceftriaxone (20 mg/kg) against *N. gonorrhoeae,* WHO-P reference strain in *G. mellonella* larvae. The test groups were injected with 2.7 x 10^7^ CFU/ml of Ng, WHO-P strain in PBS followed by (a) Lcn Z (doses -32 and 64 µg/ml), (b) GarKS (doses -32 and 64 µg/ml) or ceftriaxone (20mg/Kg). The negative control group was injected with 30 µL of PBS, and the positive control group was injected with 2.7 x 10^7^ CFU/ml of Ng in PBS. Both the test and control groups consisted of 20 larvae and were incubated for 120 hours at 37°C. Error bars represent the standard errors. Asterisks represent the significance levels based on the *p*-values comparing the survival curves between the PBS positive controls and each treatment arm (Mantel Cox test). Four (****) asterisks represent a p-value of <0.0001.
